# Efficacy Evaluation of Ciprofloxacin-Loaded Poly (Trimethylene Carbonate) Implants in the Treatment of Chronic Osteomyelitis

**DOI:** 10.3389/fbioe.2022.864041

**Published:** 2022-04-08

**Authors:** Yixiu Liu, A. Liang, Xu Li, Zhihe Ma, Dan Zhang

**Affiliations:** ^1^ Department of Orthopaedics, The Central Hospital Affiliated to Shenyang Medical College, Shenyang, China; ^2^ Shenyang Clinical Research Center for Hand and Foot, Shenyang, China; ^3^ The First People’s Hospital of Shenyang, Shenyang, China; ^4^ Liaoning Provincial Key Laboratory of Oral Diseases, School and Hospital of Stomatology, China Medical University, Shenyang, China

**Keywords:** poly(trimethylene carbonate), ciprofloxacin, chronic osteomyelitis, antibacterial activity, efficacy evaluation

## Abstract

In this study, poly (trimethylene carbonate) (PTMC) with excellent biocompatibility was synthesized via ring-opening of TMC to prepare the Ciprofloxacin-loaded PTMC implants, and antibacterial effects *in vitro* or *in vivo* of the resulting implants were investigated to evaluate the potential for treating chronic osteomyelitis. The *in vitro* results showed the Ciprofloxacin-loaded PTMC implants could sustain release ciprofloxacin at a release amount of about 90 μg/d for 28 days and possessed excellent antibacterial effect, as evidenced by the smaller size of the antibacterial ring of 32.6 ± 0.64 mm and the biofilm inhibition of 60% after 28 days of release. The *in vivo* results showed that after 28 days of treatment, the body weight and the white blood cell counts of chronic-osteomyelitis-model rats in the treatment group reached 381.6 ± 16.8 g and (7.86 ± 0.91) ×10^9^/L, respectively, returning to normal rapidly compared with the control and blank group, indicating the remarkable antibacterial effect of the Ciprofloxacin-loaded PTMC implants. X-ray images and HE staining results also confirmed that most of the proximal and middle parts of the tibia returned to typical structures and new and trabecular bone had been formed for the rats in the treatment group, and no inflammatory cells were found as compared to the control and blank groups, after 28 days of treatment. The significant lower number of colonies of (9.92 ± 1.56) × 10 CFU/g in the treatment group also suggests that the Ciprofloxacin-loaded PTMC implants achieve a practical antibacterial effect through a local application.

## Introduction

Osteomyelitis is inflammation of the bones caused by infectious microorganisms. Chronic osteomyelitis is difficult to cure and easy to relapse, bringing significant psychological and economic burden to patients (Barakat et al., 2019). Due to the severe damage to the local blood supply of the lesion, it is difficult for the blood to carry antibiotics to the infected area by oral or intravenous antibiotics, and the effective bactericidal concentration cannot be reached, which makes the infection remain and the recurrence rate is high. Toxic and side effects damage vital organs of the body, and the emergence of drug resistance quickly occurs after repeated administration of large doses (Fantoni et al., 2019).

With the development of medical and health technology, slow-release carriers loaded with antibiotics are gradually applied to treat osteomyelitis. Compared with traditional treatment methods, the sustained-release carrier has the characteristics of releasable antibiotics and absorbability. Based on controlling infection, it avoids secondary surgery to remove, and there is no need to wait for infection control before performing secondary surgery. At present, the carriers of antibiotic sustained-release systems can be mainly divided into two categories: non-biodegradable and biodegradable (Liu et al., 2021; Wassif et al., 2021). The non-biodegradable carrier is polymethylmethacrylate (polymethylmethacrylate, PMMA) (van Vugt et al., 2019; Patel et al., 2021). It is the first carrier to treat chronic osteomyelitis. With further experimental and clinical research development, PMMA gradually affects clinical application due to its non-degradable properties and the need to remove after infection control (van Vugt et al., 2019). Biodegradable carriers mainly include calcium sulfate, polylactic acid, PLGA, etc. (Cheng et al., 2018; Nabipour et al., 2018; Andreacchio et al., 2019; Zhao et al., 2020; e Silva et al., 2021). Among them, the long-term sustained-release effect of the drug delivery system based on PLA and PLGA is ideal, and it is more suitable for treating chronic osteomyelitis [ (Nabipour et al., 2018; e Silva et al., 2021; Cheng et al., 2018)]. However, the characteristic of PLA or PLGA to produce acidic degradation products during the degradation process is unavoidable (Zaaba and Jaafar, 2020; Jin et al., 2021; Ko et al., 2021), which can easily have side effects on drug activity or cause the pH of the application site to decrease, inducing sterile inflammation affects the therapeutic effect. Therefore, selecting carrier materials for biodegradable drug delivery systems is crucial for treating chronic osteomyelitis. Poly (trimethylene carbonate) (PTMC) is a polymer with excellent biocompatibility and good degradation properties, which has great potential in the fields of drug sustained release and tissue engineering (Dienel et al., 2019; Mohajeri et al., 2020; Weisgrab et al., 2020; Brossier et al., 2021). More importantly, Yang et al. show that PTMC does not generate acidic degradation products during the degradation process (Yang et al., 2014; Yang et al., 2015; Yang et al., 2016; Hou et al., 2017; Hou et al., 2019; Cai et al., 2021; Hou et al., 2021) and is an ideal carrier material for biodegradable long-acting sustained-release implants (Yang et al., 2012; Yang et al., 2013).

Ciprofloxacin has been demonstrated to be effective against a broad spectrum of bacteria associated with osteomyelitis and is an effective drug for treating osteomyelitis (Lin et al., 2019). In this study, ciprofloxacin hydrochloride, a commonly used drug for treating osteomyelitis, was used as a drug model, and PTMC was used as a drug carrier to construct a biodegradable long-term drug delivery system. The release and antibacterial properties of the Ciprofloxacin-loaded PTMC implants were investigated *in vitro*. Furthermore, a rat model of chronic osteomyelitis was established to investigate the *in vivo* antibacterial effect of PTMC implants, with the aim to verify the feasibility of PTMC implants loaded with Ciprofloxacin in the treatment of chronic osteomyelitis.

## Materials and Methods

### Materials

Trimethylene carbonate (TMC) was purchased from Daigang Co., Ltd. (Jinan, Shandong, China). Sn(Oct)_2_ (95%) was purchased from Sigma-Aldrich and used as received. Ciprofloxacin HCl was purchased from Dalian Meilun Biotechnology Co., Ltd.; Staphylococcus aureus was purchased from Shanghai Benoy Biotechnology Co., Ltd.; SPF Wistar rats were purchased from Liaoning Changsheng Biotechnology Co., Ltd. Cell Counting Kit-8 was purchased from Shanghai Aladdin Biochemical Technology Co., Ltd.

### Synthesis of PTMC

PTMC was synthesized via the ring-opening polymerization (ROP) of TMC using Sn(Oct)_2_ (1/5000 eq) as a catalyst, according to the reference (Hou et al., 2021). Briefly, the TMC monomer and the catalyst were accurately weighed and placed in an ampoule. The ampoules were purged with dry nitrogen and then heat-sealed under vacuum. Then the ampoule was immersed into an oil bath at a preset temperature of 130 ± 2°C for 24 h. After the reaction, the ampoules were cooled to room temperature and smashed to obtain the polymer. The crude oligomers were purified using ice-methanol and dried under vacuum at 37°C to constant weight.

### The Biocompatibility of PTMC

The extracts of PTMC were used for the *in vitro* cell proliferation and cytotoxicity tests. PTMC samples were immersed in an *α*-DMEM medium with 10% fetal bovine serum for 72 h at 37°C in a humidiﬁed atmosphere of 5% CO_2_. The immersion ratio was 0.1 g/ml according to ISO 10993 Part 12. *α*-DMEM medium with 10% fetal bovine serum is also immersed in the same volume for 72 h under the same conditions. The extracts and *α*-DMEM medium were ﬁltrated and collected.

The cytotoxic effects and proliferation ability of the PTMC to MC3T3-E1 cells were evaluated using the CCK8 assay. MC3T3-E1 cells were seeded in 96-well culture plates at a 1.5 × 104 cells/ml density. 100μL extracts were added in each well of the PTMC group, 100 μL *α*-DMEM medium was added in each control group well. After incubation for 24, 48 and 72 h, respectively, the PTMC group and the control group were rinsed with phosphate buffer solution (PBS) for one time. Then the PTMC group and control groups were then observed under a ﬂuorescence microscope (Nikon, Japan). The cytotoxicity evaluation was conducted by 10% (v/v) concentration of CCK-8 reagent. The spectrophotometric absorbance was measured at 450 nm using a microplate reader (Infinite M200, Tecan, Austria). Relative growth rate (RGR) was also used to evaluate the biocompatibility of PTMC. The formula for calculating RGR was RGR = ODe/ODc × 100%. ODe is the average OD value of the experimental groups. ODc is the average OD value of the control group. The cell toxicity grade (CTG) is obtained according to the standard United States Pharmacopeia. A material is considered non-toxic when the sample has an RGR value greater than 80 and a CTG rating of 0 or one according to the standard.

### Preparation and *in vitro* Release of Ciprofloxacin-Loaded PTMC Implants

Precisely weigh 5 g of PTMC, dissolve it in chloroform, and add 0.5 g of ciprofloxacin hydrochloride in a weight ratio of 1:10 of drug and carrier. The mixture was vortexed and poured into a PTFE dish. After the chloroform had evaporated entirely, the films were peeled off and dried to constant weight. Then the film was cut into small pieces and extruded into cylindrical implants (od = 1.5 mm, length = 2 cm) with a hot-melt extruder at 180 °C. After manufacture, the implants were packed in sealed bags and then irradiated with 25 kGy of 60Co for sterilization.

The PTMC implants loaded with Ciprofloxacin HCL were immersed in a glass container filled with 10 ml of PBS solution (pH = 7.4) and shaken at a frequency of 65 times/min in a constant temperature air bath at (37 ± 1) °C. The PBS solutions were changed every 24 h. Then, the replaced PBS solution was filtered through a 0.22 μm nylon membrane filter, and 2 μL of the filtrate was injected into the UHPLC system, using a mixture of 0.025 mol/L phosphoric acid solution (pH = 3.0 ± 0.1)-acetonitrile (75: 25) as mobile phase, at a flow rate of 0.1 ml/min to measure Ciprofloxacin concentration in each sample at 277 nm. The procedure was performed in triplicate for each time point, and the results were expressed as mean ± standard deviation.

### Antibacterial Activity *in vitro* of Ciprofloxacin-Loaded PTMC Implants

The activated Staphylococcus aureus was appropriately diluted with PBS solution to obtain a 10^6^ CFU/ml bacteria solution. 0.1 ml of Staphylococcus aureus bacterial solution was pipetted and evenly inoculated into a nutrient agar Petri dish. Holes were punched on the nutrient agar plate in the dish, and 0.2 ml of PTMC implants release solution from days 1, 7, 14 and 28 were added. Then, the dish was incubated at 37°C for 24 h. Then, the diameter of the bacteriostatic zones was measured accurately. The procedure was performed in triplicate, and the results were expressed as mean ± standard deviation. Staphylococcus aureus was also incubated in the 25 mg/ml leaching solution of the blank PTMC implants.

After 1, 7, 14 and 28 days of the PTMC implants release, 90 μL of the release solution was taken into a 96-well plate, added with 10 μL of TSB solution containing S. aureus (approximately 1 × 10^6^ CFU/μL) and incubated at 37°C for 24 h. Then the medium was removed, and each well was washed with 100 µL of PBS for three times, stained with 100 μL of 1% (w/v) crystal violet in water for 15 min, and then rinsed with demineralized water. 100 µL of ethanol/acetone (80:20) was added to each well to dissolve the crystal violet, and the absorbance of the crystal violet solution was measured at a wavelength of 575 nm. The absorbance (A_1_) of crystal violet solution is proportional to the biofilm amount grown in each well, and the biofilm inhibition was calculated according to the following equation:

$$\text{biofilm inhibition}=\frac{A-A_{1}}{A}\times 100\%$$

where A was the absorbance of the crystal violet solution for the PTMC implants without Ciprofloxacin.

### Establishment and Treatment of Chronic Osteomyelitis Model

The chronic osteomyelitis model was established on Wistar rats according to Karau et al. (Karau et al., 2013), and the Animal Ethics Committee of The Central Hospital Affiliated to Shenyang Medical College approved all the surgical procedures. Twenty-four rats were weighed and anaesthetized by intraperitoneal injection of sodium pentobarbital (50 mg/kg). The proximal 1/3 of the tibia of rats was exposed by the skin incision of 1.5 cm made under the right knee along the medial anterior tibial condyle. Then the bone marrow cavity of the tibia was exposed using an electric drill with a diameter of 1.5 mm, rinsed with saline, injected with 50 μL of morrhuate sodium injection, and 50 μL sterile saline of Staphylococcus aureus was injected. After that, the bone marrow cavity was closed with bone wax and sutured. After surgery, the rats were caged and allowed to move freely and eat regularly. Four weeks later, six rats were sacrificed, and the formation of chronic osteomyelitis was confirmed by X-ray (Xie et al., 2019) and HE staining (Musher and Arasaratnam, 2021).

Eighteen model rats of chronic osteomyelitis were randomly divided into a treatment group, a control group, and a blank group, with six rats in each group. The model rats were anaesthetized by intraperitoneal injection of sodium pentobarbital (50 mg/kg). Then the bone marrow cavity was exposed and opened again through the original incision with a 1.5 mm diameter electric drill for debridement to remove necrotic, sclerotic, and infected tissues, and then repeatedly flushed with 2% hydrogen peroxide and saline. The treatment group was given a 2 cm Ciprofloxacin-loaded PTMC implant, the control group was assigned a 2 cm PTMC implant without Ciprofloxacin, and the blank group has received no treatment. Afterwards, the marrow cavity was closed with bone wax and sutured. The body weight and the white blood cell (WBC) counts of rats in the three groups were observed on the 0, 7, 14, 21, and 28 days after the treatment. After 28 days of treatment, all the rats were sacrificed, and the right tibia specimen was taken. Three for HE staining and three for X-ray analysis and then pulverized into powder. 1 g of bone powder was accurately weighed and added into 1 ml of physiological saline to make a suspension. After tenfold serial dilution with saline, 0.1 ml of the diluted sample was inoculated onto blood agar plates and incubated at 37°C for 48 h to count the number of bacterial colonies. All tests were carried out in triplicate and under aseptic conditions.

## Results and Discussion

### Synthesis of PTMC

**FIGURE 1:**
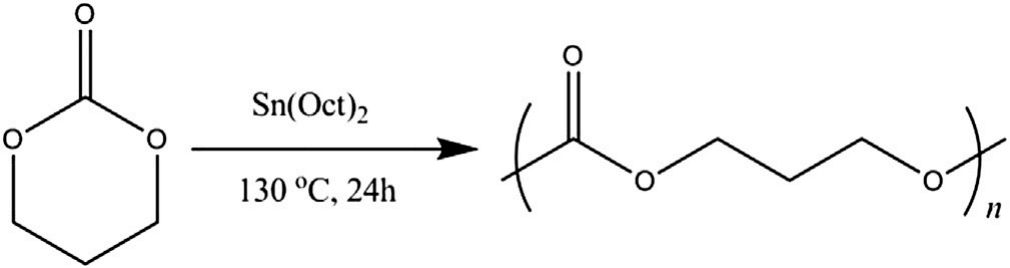
The synthetic route of PTMC.

PTMC was synthesized by bulk ring-opening copolymerization of trimethylene carbonate monomer in the presence of SnOct_2_ as a catalyst at 130°C, as illustrated in Figure 1. The structure of PTMC was determined by 1H NMR [(δ, ppm from TMS in CDCl3): 2.02 (-CH_2_--C ***H***_2_--CH_2_-) and 4.14 (-OC ***H***_2_--CH_2_-)]. The molecular weight and polydispersity of PTMC were determined using GPC, showing a polydispersity of 1.07 and a molecular weight of 3.29 × 10^5^ g/mol. The intrinsic viscosity of the resulting PTMC was 6.54 dl/g. The thermal properties of the PTMC were determined using DSC and TGA. The TGA thermogram of PTMC showed that the polymer began to degrade at 267.0 °C, and the DSC studies showed that the PTMC polymer was amorphous with only one glass transition temperature of -13.9 °C. The water contact angle results show that the obtained PTMC was hydrophobic with a static water contact angle of 87.25 ± 0.92^°^.

### The Biocompatibility of PTMC

**FIGURE 2:**
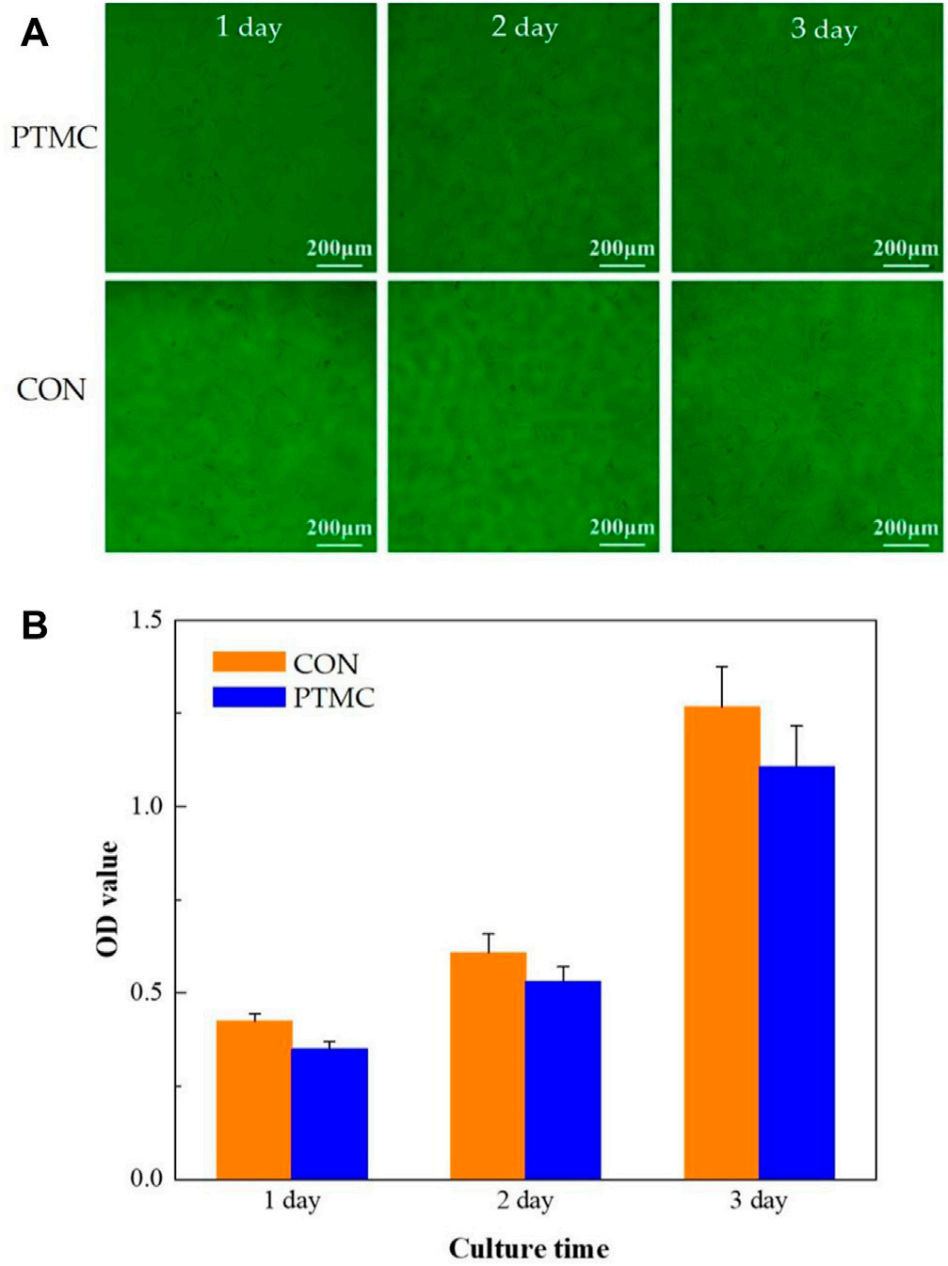
The morphology **(A)** and proliferation **(B)** of MC3T3-E1 cells cultured in PTMC extracts.

In this study, the biocompatibility of PTMC was investigated using cell proliferation and cytotoxicity tests. The MC3T3-E1 cells cultured in the well with PTMC for 24, 48 and 72 h are shown in Figure 2A. The results show that cells grew well in solutions containing PTMC extracts. Compared with the control group, there are no significant differences in the number and morphology of cells in the PTMC group. Figure 2B shows the optical densities of MC3T3-E1 cells in PTMC extracts measured by the CCK8 test. It could be seen that the optical density (OD) values of all the groups increased gradually with increasing cultivation time.

The relative growth rates (RGR) of MC3T3-E1 cells in PTMC extracts are shown in Table 1. From 24 to 72 h of incubation, the average RGR value was greater than 80%, and all the experimental groups showed grade 1. The cell proliferation assay results showed no significant difference between the groups, indicating that PTMC has excellent biocompatibility and would not be deleterious to cell viability. The result was similar to that of Papenburg’s report (Papenburg et al., 2009), which also revealed the high cell attachment and proliferation of PTMC.

**TABLE 1:** The relative growth rates (RGR) and cell toxicity grade (CTG).

Time (h)	RGR	CTG
24	82.48% ± 0.04	1
48	87.24% ± 0.05	1
72	87.47% ± 0.09	1

### *In vitro* Release of Ciprofloxacin-Loaded PTMC Implants

**FIGURE 3:**
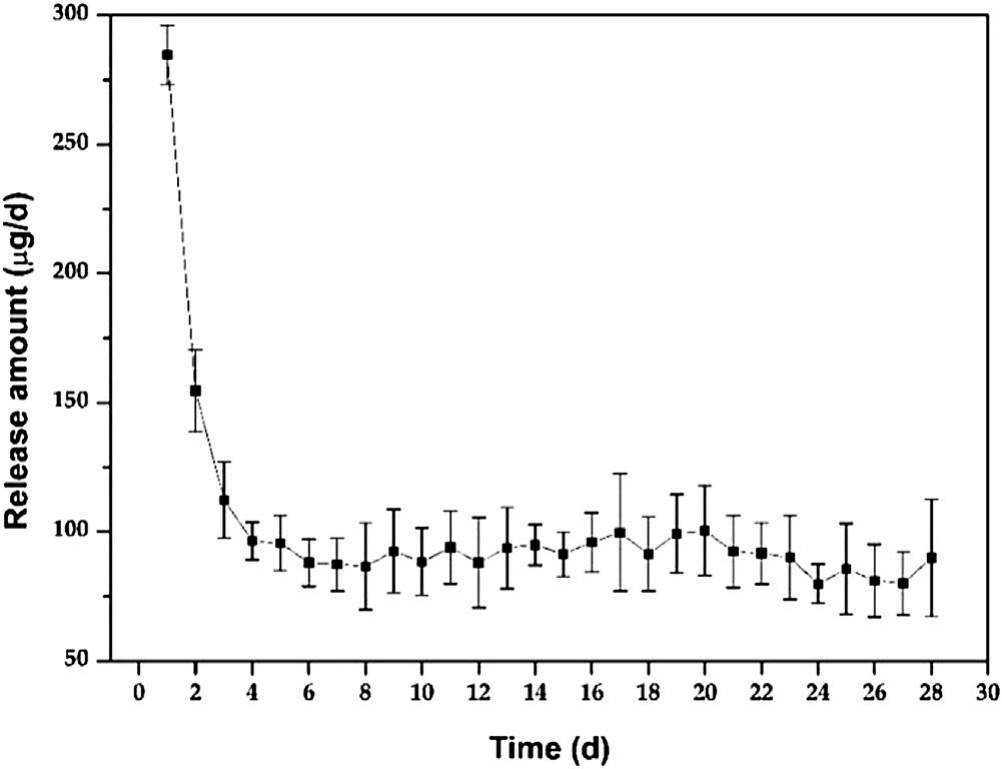
*In vitro* release of the Ciprofloxacin-loaded PTMC implants in PBS solution.

**FIGURE 4:**
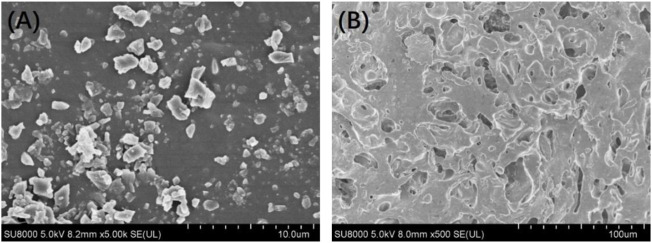
Surface morphology of the ciprofloxacin-loaded PTMC implants before **(A)** and after **(B)** release in PBS solution.

Figure 3 shows the *in vitro* release behaviour of the Ciprofloxacin-loaded PTMC implants. The relatively high release rate indicates a “burst release” occurred in the early stage of the *in vitro* release. Apparently, the average release amount was 284.7 ± 11.6 μg/d at day 1. It was attributed to the preferential release of Ciprofloxacin enriched on the surface of PTMC implants, as evidenced by the holes displayed on the SEM of the implant surface (Figure 4). As shown in Figure 4, ciprofloxacin particles were distributed on the surface of PTMC implants before release, while the holes rather than particles were seen after release.

After the “burst release”, the release of PTMC implants decreased gradually to 96.4 ± 7.2 μg/d at day 4, and then the release tended to be gentle, with an average release amount of 89.9 ± 12.6 μg/d on day 28. The result indicates that the Ciprofloxacin concentration released from PTMC implants exceeded the minimum inhibitory concentration of Ciprofloxacin (0.4 μg/ml) and was sufficient for osteomyelitis treatment (Mirzaie et al., 2020). The “burst release” in the early stage of *in vitro* release helps to quickly inhibit inflammation, while the sustained and stable release of ciprofloxacin hydrochloride in the later stage helps to control inflammation and consolidate the anti-inflammatory effect effectively. Hence, the synergistic effect of early “burst release” and late stable, sustained release of PTMC implants is ideal for treating chronic osteomyelitis.

Furthermore, the release behaviour of PTMC implants was similar to that of poly (d, l-lactide-co-glycolide-co-ε-caprolactone) (PLGC) reported in our previous work (Liu et al., 2020). However, unlike PLGC, there is no second “burst release” caused by autocatalytic degradation of the acidic degradation products was observed for PTMC implants, resulting in much more stability of the PTMC implants and much more controllable to the release of ciprofloxacin HCL.

### Antibacterial Effect *in vitro* of Ciprofloxacin-Loaded PTMC Implants

**FIGURE 5:**
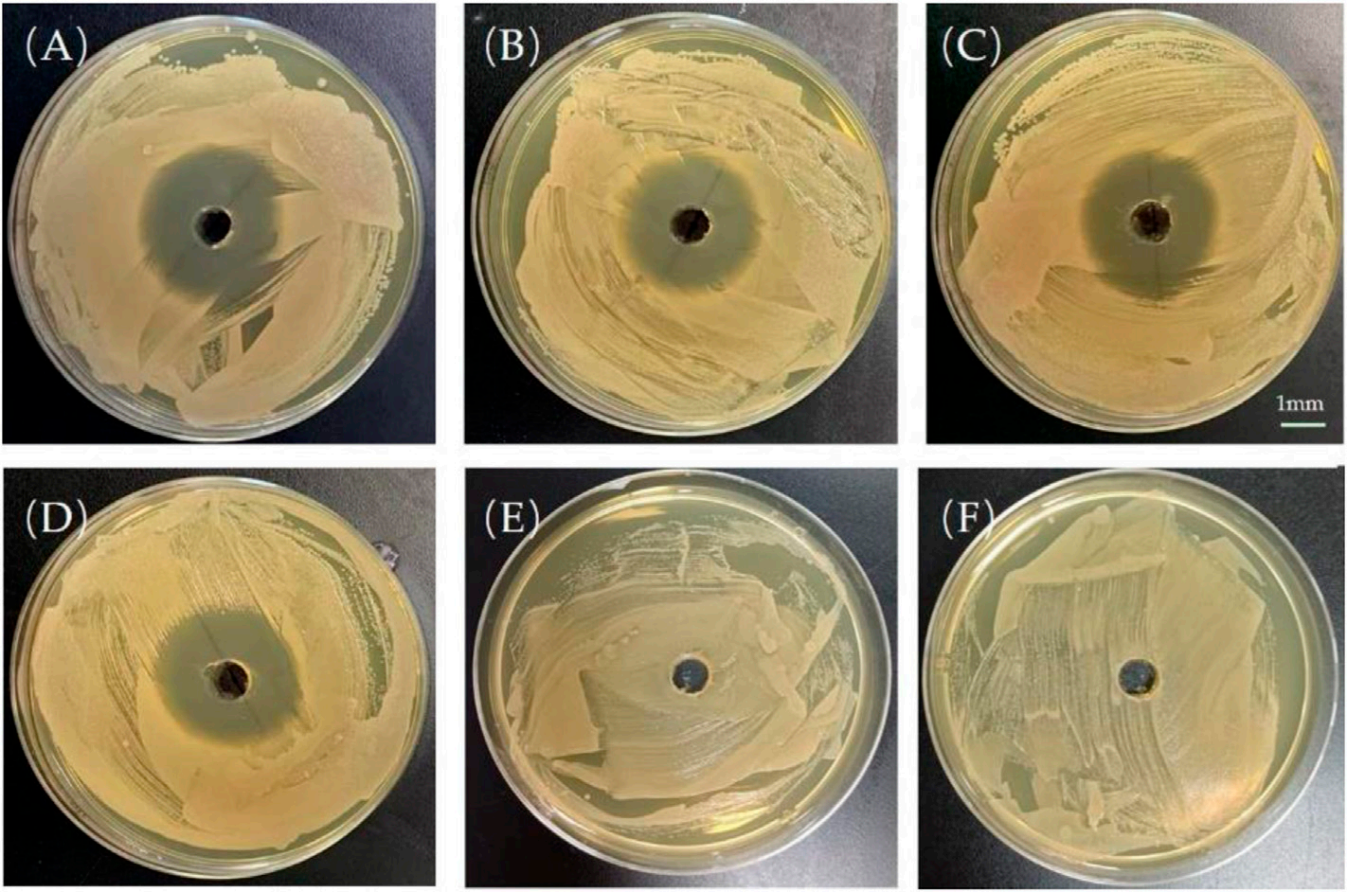
Bacteriostatic rings produced by the release solution of the ciprofloxacin-loaded PTMC implants at day 1 **(A)**, day 7 **(B)**, day 14 **(C)**, day 28 **(D)**; Bacteriostatic rings produced by the leaching solution of blank PTMC at day 1 **(E)** and day 7 **(F)**. The scale bar is 1 mm.

For a more direct measurement of the antimicrobial efficacy of PTMC implants *in vitro*, a semi-quantitative evaluation of the antibacterial effect was performed by measuring the diameter of the antibacterial ring (Figure 5). The presence of the bacteriostatic ring indicates that Ciprofloxacin was biologically active in PTMC implants. As shown in Figure 3, the size of the antibacterial ring was 38.7 ± 0.52 mm for the release solution after 1 day of release of PTMC implants. The antibacterial ring sizes of the released solutions after 7, 14 and 28 days of release were 30.6 ± 3.21 mm, 33.3 ± 1.52 mm, and 32.6 ± 0.64 mm, respectively. The results confirm that Ciprofloxacin-loaded PTMC implants could inhibit the growth of S. aureus for up to 28 days. No bacteriostatic rings were presented for the blank PTMC group, indicating that PTMC has no antibacterial effect.

**FIGURE 6:**
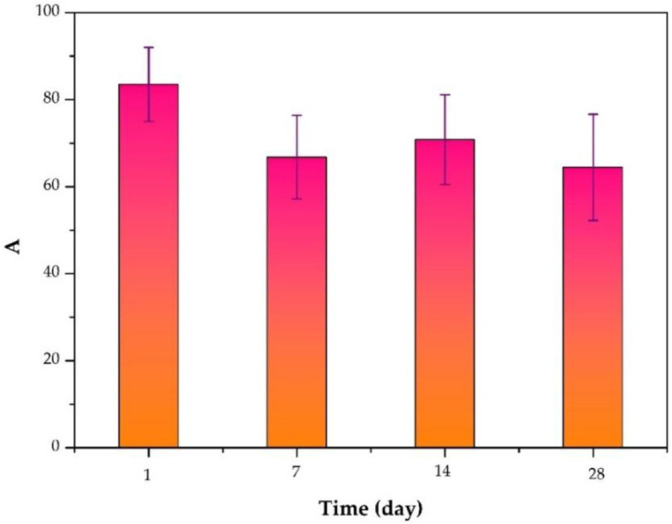
Inhibition of S. aureus biofilm formation by the release solution of the ciprofloxacin-loaded PTMC implants at day 1, day 7, day 14, and day 28.

The inhibition of S. aureus biofilm formation by the release solution of the ciprofloxacin-loaded PTMC implants at day 1, day 7, day 14, and day 28 (d) is presented in Figure 6, and the inhibition was 83.50 ± 8.52%, 66.77 ± 9.65%, 70.81 ± 10.34%, 64.44 ± 12.26%, respectively. The inhibition efficiency was proportional to the release concentration of ciprofloxacin. The higher the release concentration of ciprofloxacin (Figure 3), the higher inhibition efficiency (Figure 6), and the larger the diameter of the bacteriostatic zone (Figure 5). The results confirmed that after 28 days, the ciprofloxacin-loaded PTMC implants could still inhibit Staphylococcus aureus biofilm formation by more than 60%, indicating that the delivery system has an excellent antibacterial effect.

### Establishment and Treatment of Chronic Osteomyelitis Model Rats

**FIGURE 7:**
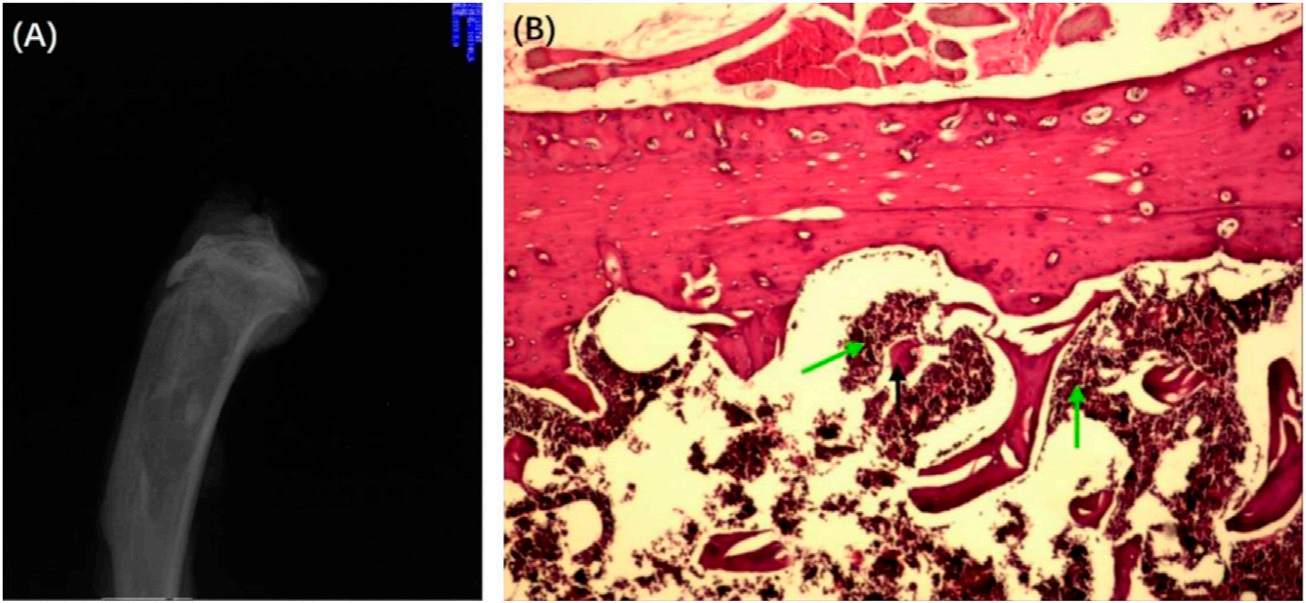
X-ray **(A)** and HE-stained section **(B)** of the chronic osteomyelitis model. Black arrows: deadbone; green arrows: inflammatory cells.

To honestly evaluate the antibacterial effect *in vivo*, the Wistar rat model of chronic osteomyelitis was first established in Wistar rats. The successful establishment of chronic osteomyelitis was confirmed by X-ray imaging (Figure 7A). As shown in Figure 7A, the tibia of all rats showed apparent signs of osteomyelitis, such as marked reduction of bone density, thinning or even disappearance of trabecular bone, damage of cortical bone, and formation of sequestrum. Other signs such as a widening of the proximal medullary cavity and periosteal reactions were also observed in Figure 7A. The successful establishment of chronic osteomyelitis was also confirmed by HE observation (Figure 7B). As shown in Figure 7B, many inflammatory cells, mainly plasma cells and lymphocytes, were seen in the microcavity lesions, and sequestrum formation and fibrous tissue proliferation were seen in HE sections, indicating the formation of osteomyelitis.

After the formation of osteomyelitis, the rat model was treated as follows according to the group, the treatment group was given a Ciprofloxacin-loaded PTMC implant, the control group was assigned a PTMC implant without Ciprofloxacin, and the blank group has received no treatment. Observation of physical signs showed that the mobility of the treatment group was significantly better than that of the other two groups. Seven days after implantation of PTMC implants, the swelling of the surgical site of the rats in the treatment group disappeared, and the walking was more flexible than before. Fourteen days after implantation, the wound at the surgical site of the rats was healed entirely, and the gait returned to normal.

**FIGURE 8:**
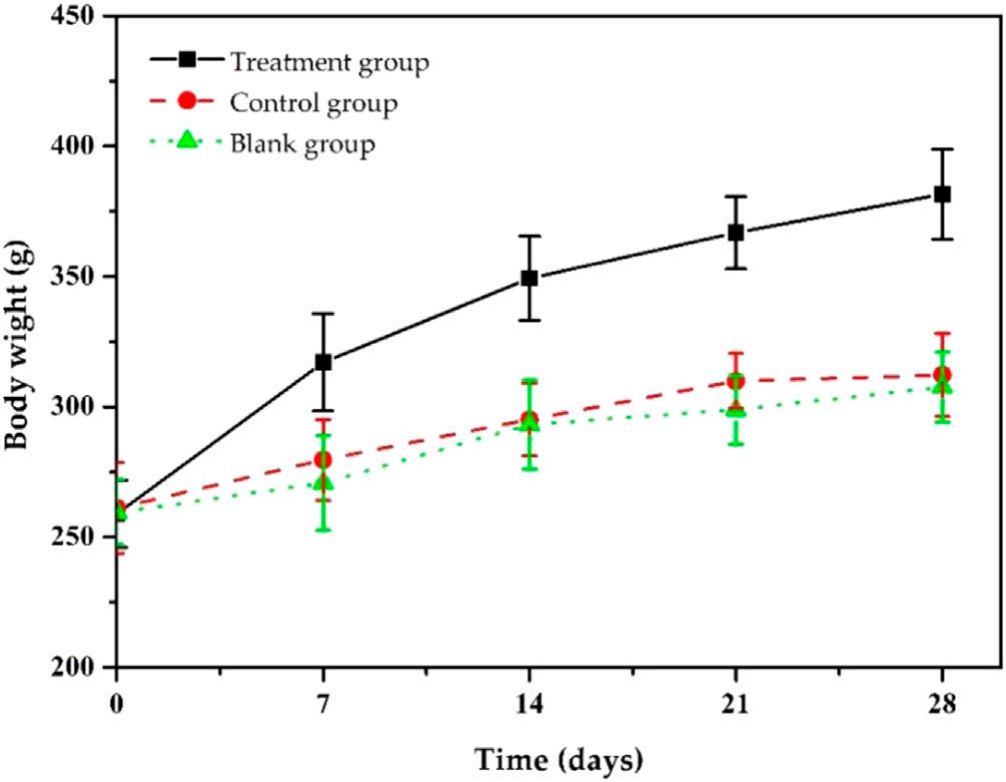
Changes in body weights of chronic osteomyelitis model rats during treatment.

During the treatment, we assessed the body weight changes of the model rats (Figure 8). As shown in Figure 8, after 7 days of treatment, the bodyweight of the rats in the treatment group gradually increased, and after 14 days, the bodyweight of the rats returned to normal and reached the preoperative value. After 28 days of treatment, their body weight increased to 381.6 ± 16.8 g. The weight gain in the treatment group was thought to be due to increased appetite led by reduced inflammation. Rats in the control and blank groups gained to 312.3 ± 15.9 g and 307.6 ± 13.5 g, respectively, lower than that of the treatment group because they did not significantly reduce inflammation due to insufficient antibiotic treatment. The body weight changes in each group indicate that our ciprofloxacin-loaded PTMC implants had a significant antibacterial effect.

**FIGURE 9:**
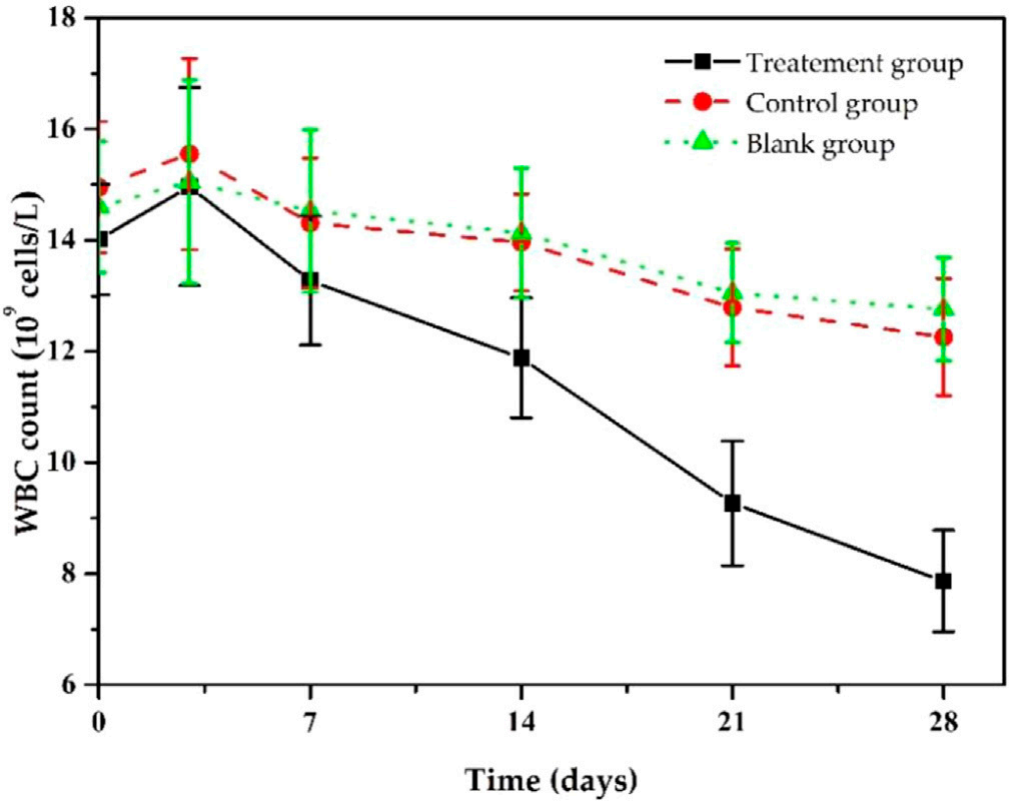
Changes in white blood cell counts s of chronic osteomyelitis model rats during treatment.

At the same time, we also monitored the changes in the rats’ white blood cell (WBC) counts in each group during the treatment (Figure 9). Before implantation, WBC counts of the rats in the treatment group was (14.01 ± 0.99)×10^9^/L, significantly higher than the standard value, resulting from chronic bacterial inflammation. Due to postoperative stress response, it further increased to (14.96 ± 1.65)×10^9^/L after 3 days of implantation, then it decreased slowly and returned to an average value of about (7.86 ± 0.91) ×10^9^/L after 28 days. The trends in WBC counts in the control and the blank group were similar, but the downward trend was much slower, and WBC counts were (12.56 ± 1.05) ×10^9^/L and (12.76×±0.93) ×10^9^/L, respectively, at day 28, higher than that of treatment group. The statistical results showed that after 28 days of treatment, the WBC changes between the treatment group and the other groups were statistically significant (*p* < 0.05), while there was no significant difference between the blank and control groups (*p* > 0.05). These results indicate that Ciprofloxacin-loaded PTMC implants have significant antibacterial efficacy.

**FIGURE 10:**
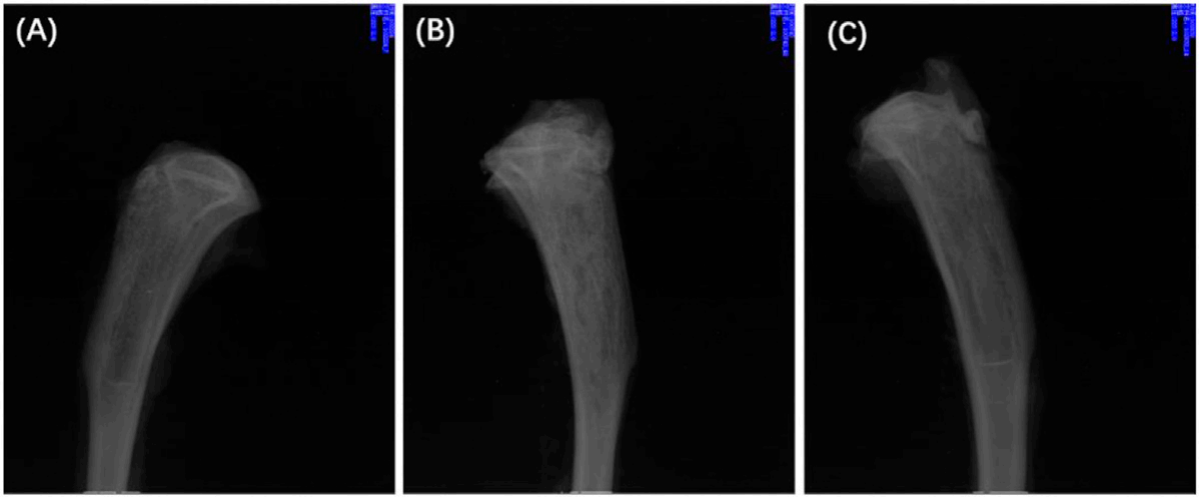
X-rays of chronic-osteomyelitis-mode. treatment group **(A)**, control group **(B)** and blank group **(C)** after 28 days of treatments.

Twenty-eight days after treatment, the X-ray imaging of the model rats in each group was determined (Figure 10). The radiographic results show that most of the proximal and middle parts of the tibia in the treatment group returned to typical structures, and no bone destruction and periosteal reaction were found (Figure 10A), while the signs as mentioned above of osteomyelitis were still found in the control group, and blank group (Figures 10B, C). The results confirmed the effective antibacterial activity of the Ciprofloxacin-loaded PTMC implants *in vivo*.

**FIGURE 11:**
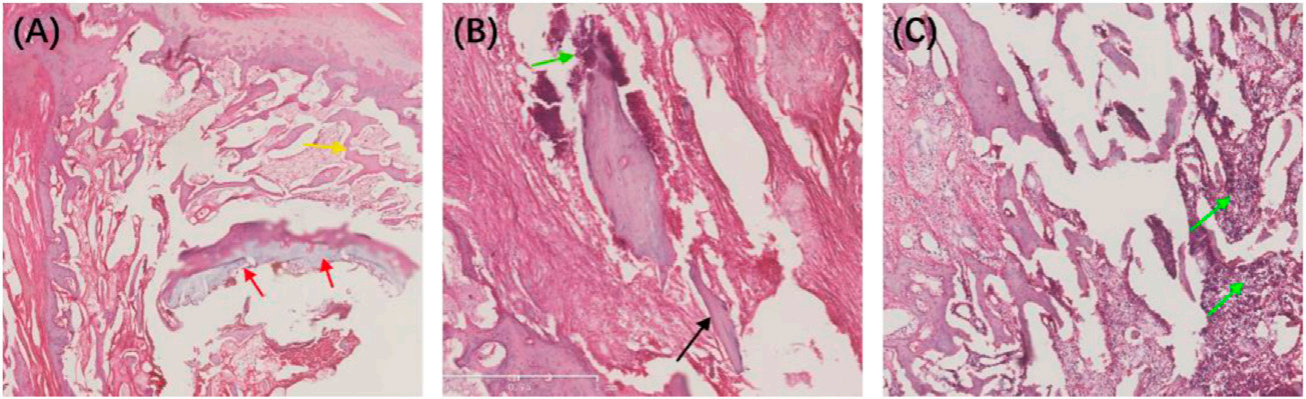
HE-stained sections of the chronic-osteomyelitis-mode. Treatment group **(A)**, control group **(B)** and blank group **(C)** after 28 days of treatments. Red arrow, new bone; yellow arrow, trabecular bone; black arrow, dead bone; green arrow, inflammatory cells.

We also performed HE staining analysis on the tibia tissue of the model rats in each group after treatment. The results showed that the new and trabecular bone in the treatment group had been formed, and no inflammatory cells were found (Figure 11A). However, the dead bone and inflammatory cells were clearly visible in the control and blank groups (Figures 11B, C). These results directly indicate that the Ciprofloxacin-loaded PTMC implants can eliminate bacterial infections.

Finally, we evaluated the therapeutic efficacy of the Ciprofloxacin-loaded PTMC implants in treating chronic osteomyelitis using a microbiological test, and the results were expressed as bacterial colonies per Gram of bone tissue (Table 2). The results showed that the number of colonies in the treatment group was (9.92 ± 1.56)×10^1^ CFU/g, while that in the control and blank groups were (6.37 ± 0.31)×10^5^ and (6.70 ± 0.38)×10^5^, respectively. Obviously, the number of colonies in the treatment group was significantly lower than in the other groups. The statistical results showed that the difference between the treatment group and the other groups was statistically significant (*p* < 0.05), while the difference between the control group and the blank group was not statistically significant (*p* > 0.05). These results suggest that the Ciprofloxacin-loaded PTMC implants achieve a practical antibacterial effect through a local application.

(CFU/g, x ± s, n =3)

**TABLE 2:** Counts of bacterial content in each group of bone tissue after 28 days of treatment.

No	Treatment Group	Control Group	Blank Group
1	8.2×10^1^	6.3×10^5^	6.6×10^5^
2	8.8×10^1^	6,6×10^5^	6.2×10^5^
3	10.0×10^1^	6.2×10^5^	6.9×10^5^
4	9.6×10^1^	6.3×10^5^	6.4×10^5^
5	10.2×10^1^	6.5×10^5^	6.9×10^5^
6	12.7×10^1^	6.9×10^5^	7.2×10^5^
Average	(9.92 ± 1.56)×10^1^	(6.37 ± 0.31)×10^5^	(6.70 ± 0.38)×10^5^

## Conclusion

To overcome the disadvantages of non-degradable PMMA and acidic degradation products of PLGA in the existing local treatment methods for osteomyelitis, PTMC was synthesized as the carrier to fabricate a ciprofloxacin-loaded PTMC implant in this study to develop a new strategy for the treatment of chronic osteomyelitis. The antibacterial effect and the therapeutic effect of the resulting system on chronic osteomyelitis were investigated.

*In vitro*, drug release results and antibacterial effects such as antibacterial rings and biofilms inhibition suggest that ciprofloxacin-loaded PTMC implants can sustainably release Ciprofloxacin HCL at sufficient concentrations and maintain antibacterial effects for 28 days. *In vivo*, treatment of chronic osteomyelitis model rats showed the considerable antibacterial effect of the ciprofloxacin-loaded PTMC implants, as evidenced by the gradual return of rat body weight and WBC counts to pre-implantation levels. X-ray imaging, HE staining, and lower bacterial colonies per Gram of bone tissue also confirmed the unique antimicrobial effect of ciprofloxacin-loaded PTMC implants. Through *in vitro* and *in vivo* antibacterial results, this study confirmed the great potential of the ciprofloxacin-loaded PTMC implants in treating chronic osteomyelitis.

## Data Availability

The raw data supporting the conclusion of this article will be made available by the authors, without undue reservation.
